# Women with Metabolic Syndrome and General Obesity Are at a Higher Risk for Significant Hyperuricemia Compared to Men

**DOI:** 10.3390/jcm8060837

**Published:** 2019-06-12

**Authors:** In Young Kim, Kyung-Do Han, Da Hye Kim, Yeonghee Eun, Hoon-Suk Cha, Eun-Mi Koh, Jaejoon Lee, Hyungjin Kim

**Affiliations:** 1 Department of Medicine, National Police Hospital, 123, Songi-ro, Songpa-gu, Seoul 05715, Korea; ariatansi@naver.com; 2 Department of Biostatistics, College of Medicine, The Catholic University of Korea, 222, Banpo-daero, Seocho-gu, Seoul 06591, Korea; hkd917@naver.com (K.-D.H.); dahaene1228@naver.com (D.H.K.); 3 Department of Medicine, Samsung Medical Center, Sungkyunkwan University School of Medicine, 81 Irwon-Ro Gangnam-gu, Seoul 06351, Korea; yeonghee.eun@samsung.com (Y.E.); hoonsuk.cha@samsung.com (H.-S.C.); eunmi.koh@samsung.com (E.-M.K.)

**Keywords:** Hyperuricemia, metabolic syndrome, general obesity

## Abstract

Hyperuricemia is an emerging potential biomarker for metabolic syndrome (MetS) and its complications. This study aimed to investigate the risk factors of hyperuricemia, particularly, the association of hyperuricemia with MetS and general obesity according to sex. We performed multivariate logistic regression analyses using the 2016 Korean National Health and Nutrition Examination Survey data. Hyperuricemia was defined by a serum uric acid level ≥7.0 mg/dL for men and ≥6.0 mg/dL for women. General obesity was based on a body mass index (BMI) ≥25 kg/m^2^. Among a total of 5591 Korean adult participants, 685 (12.3%) individuals had hyperuricemia. Hyperuricemia was significantly associated with MetS in men (odds ratio (OR): 2.38, 95% CI: 1.84–3.08) and in women (OR: 4.15, 95% CI: 2.75–6.28) after adjustments. General obesity was also independently related to hyperuricemia in both sexes (OR: 2.17, 95% CI: 1.76–2.68 in men, OR: 3.80, 95% CI: 2.82–5.12 in women). In subgroup analyses, the presence of concomitant MetS and general obesity posed a strikingly higher risk for hyperuricemia among women (OR: 7.24, 95% CI: 4.56–11.50) when compared to men (OR: 2.90, 95% CI: 2.12–3.96). More attention should be paid to the increased risk of hyperuricemia for females with both MetS and general obesity.

## 1. Introduction

Uric acid is the end product of purine metabolism, and hyperuricemia arises from the imbalance between the production and excretion of uric acid. Uric acid is produced in the liver, adipose tissue, and muscle, then it is primarily excreted by renal clearance [1,2]. Hyperuricemia may lead to notorious complications, such as gout, urolithiasis, and nephropathy. The prevalence of hyperuricemia varies according to studies from diverse countries. In a recent study based on data from the 2016 Korean National Health and Nutrition Examination Survey (KNHANES), the age-standardized prevalence of hyperuricemia (defined as a serum uric acid (sUA) level >7.0 mg/dL in men and >6.0 mg/dL in women) was 11.4% (17.0% in men and 5.9% in women) in Korea [3]. Among the general US adult population, the prevalence of hyperuricemia (sUA >7.0 mg/dL in men and >5.7 mg/dL in women) was estimated to be 21.4% in 2007–2008. They found that the prevalence had increased over the past two decades [4]. Several other studies have also demonstrated the globally increasing prevalence of hyperuricemia and gout. Westernized diet, increased longevity, increased prevalence of obesity, hypertension, or use of diuretics have been suspected to contribute to this increase [5,6,7]. 

Metabolic syndrome (MetS) refers to a constellation of interrelated potential risk factors for the development of atherosclerotic cardiovascular disease, type 2 diabetes mellitus, and increased mortality [8,9,10,11,12]. Growing evidence has suggested that hyperuricemia is correlated with metabolic and cardiovascular diseases. The association between hyperuricemia, MetS, and insulin resistance has been advocated from previous studies and sUA level has been suggested as a potential biomarker for these conditions [13,14,15,16,17,18,19,20,21,22,23,24,25,26,27,28,29,30,31,32,33,34,35,36,37,38]. Along with MetS, obesity has also been suggested as an significant risk factor for cardiovascular diseases and mortality [39,40,41,42]. Furthermore, various studies have indicated a link between obesity and hyperuricemia [43,44,45,46,47]. 

For this reason, hyperuricemia is emerging as one of the major medical issues, given the increasing prevalence and growing importance in the context of public health. Recently, Lee et al. reported that the prevalence of MetS was still increasing from 2009 to 2013 in Korea. In particular, the prevalence of central obesity (defined by raised waist circumference) among young Korean adults has significantly increased [48].

In consideration of the potential relationship between hyperuricemia, MetS, and obesity, as well as their importance implicated in the development of cardiovascular disease and increased mortality, it is necessary to better understand the correlation between these conditions. Thus, this study aimed to investigate the association of hyperuricemia with MetS and general obesity, based on the 2016 Korean National Health and Nutritional Examination Survey (KNHANES) data. We also proposed to estimate the relative risk of having hyperuricemia according to the presence of MetS, general obesity, or both conditions.

## 2. Materials and Methods

### 2.1. Study Population and Source of Data

This study was performed using the KNHANES database, a representative sample of the Korean population. We used the data of the latest KNHANES, which was conducted in 2016. Subjects had participated in health interviews and examinations, including blood sampling. Of the 8150 participants whose data were used in the 2016 KNHANES, individuals under 19 years of age and those with incomplete data were excluded from this study. As a result, a total of 5591 individuals were enrolled for this study. All participants provided written informed consent for the survey. The Institutional Review Board of Samsung Medical Center approved this study protocol (number: SMC 2018-08-176), and all methods were performed in accordance with the relevant guidelines and regulations. 

### 2.2. Definition of Hyperuricemia

Hyperuricemia was defined by a sUA level of ≥7.0 mg/dL in men and ≥6.0 mg/dL in women. 

### 2.3. Covariates

All subjects were required to complete self-administered questionnaires, which included questions pertaining to smoking, alcohol intake, physical activity, past medical history, and income level. Subjects were classified as never-smoker, ex-smoker, or current smoker, according to their smoking history. They were classified into three groups according to the amount of alcohol consumption per day during the 1-month period before the interview: Nondrinkers, light drinkers (<30 g/day), and moderate-to-heavy drinkers (≥30 g/day). Subjects who had ≥150 minutes of moderate intensity activity or ≥75 minutes of vigorous activity for 1 week were classified as physically active. Height, weight, waist circumference, systolic blood pressure (BP), and diastolic BP were obtained by a trained nurse. Waist circumference (WC), as an indicator of abdominal obesity, was measured at the narrowest point during exhalation. Body mass index (BMI) was calculated as weight in kilograms, divided by height in square meters (kg/m^2^). General obesity was defined by a BMI ≥25 kg/m^2^, regardless of sex. Venous blood samples were obtained for all subjects after overnight fasting. Diabetes was defined by fasting plasma glucose (FPG) levels ≥126 mg/dL, being on treatments with insulin or oral agents, or a diagnosis by a physician. Hypertension was defined by SBP ≥140, or DBP ≥90 mmHg, or being on an antihypertensive treatment.

### 2.4. Definition of Metabolic Syndrome (MetS)

We defined MetS based on the revised National Cholesterol Education Program Adult Treatment Panel III (NECP-ATP III) criteria and adopted an Asian-specific WC threshold, proposed by the International Diabetes Foundation (IDF) [8,9,49]. Individuals that met any three of the following five criteria were classified as having MetS: 1) Central obesity; WC ≥90 cm in men, WC ≥85 cm in women; 2) Elevated BP; systolic BP ≥130 mmHg and/or diastolic BP ≥85 mmHg or on antihypertensive medications; 3) Hyperglycemia; defined by elevated FPG ≥100 mg/dL or on antidiabetic medications; 4) Hypertriglyceridemia; defined by elevated triglyceride (TG) ≥150 mg/dL or on medication for elevated TG; 5) Reduced high-density lipoprotein cholesterol (HLD-C); HDL-C <40 mg/dL in men, <50 mg/dL in women or on medication for reduced HCL-C. 

### 2.5. Statistical Analyses

Statistical analyses were performed using the SAS survey procedure (version 9.4; SAS Institute, Cary, NC, USA) to reflect the complex sampling design. Values are presented as mean values ± standard deviation (SD) for continuous variables, and as numbers and percentages (%) for categorical variables. The distribution of TG levels was considerably skewed and was log-transformed before the statistical analysis. The t-test and chi-square test were used to discuss differences of clinical measurements. Pearson’s correlation coefficient was used to evaluate the relationship between sUA levels and variables (BMI, WC, BP, total cholesterol (TC), low-density lipoprotein cholesterol (LDL-C), HDL-C, TG, and FPG). Multivariate logistic regression analysis was performed to estimate the association of hyperuricemia with MetS, its various components, and general obesity. We calculated the adjusted odds ratio (OR) with its 95% confidence interval (95% CI). Subgroup analyses were conducted after categorizing the subjects according to age, sex, and presence of MetS with or without general obesity. The statistical tests were two-sided, and a *p* value <0.05 was considered statistically significant. 

### 2.6. Data Availability Statement

The datasets generated during the current study are available from the corresponding author upon reasonable request. 

## 3. Results

### 3.1. Baseline Characteristics of the Study Population

Among 5591 participants who enrolled in this study, a total of 685 individuals were classified as having hyperuricemia. Table 1 presents the baseline characteristics of the study population. Among the overall subjects with hyperuricemia, more than two-thirds were male and the mean age was 45.7 ± 0.8 years. Subjects with hyperuricemia had a higher body mass index (BMI), waist circumference (WC), systolic blood pressure (BP), diastolic BP, triglyceride (TG), total cholesterol (TC), and LDL-cholesterol (LDL-C), with lower HDL-cholesterol (HDL-C), and estimated glomerular filtration rate (eGFR), compared to those without hyperuricemia. The proportion of individuals with a smoking history, moderate-to-heavy alcohol intake, hypertension, and general obesity (defined by BMI ≥25 kg/m^2^) was higher in the hyperuricemia group. MetS and all of its five components demonstrated a higher prevalence among subjects with hyperuricemia. However, when we analyzed men and women separately, the prevalence of hyperglycemia was different between two groups only among women. The differences in age and percentage of subjects with overt Diabetes Mellitus (DM) between normouricemia and hyperuricemia groups showed the opposite among men and women. The proportion of subjects with smoking or alcohol consumption was higher in the hyperuricemia group only among women. The percentage of inoccupation or a low-income level was also higher only in hyperuricemic women.

**Figure 1:**
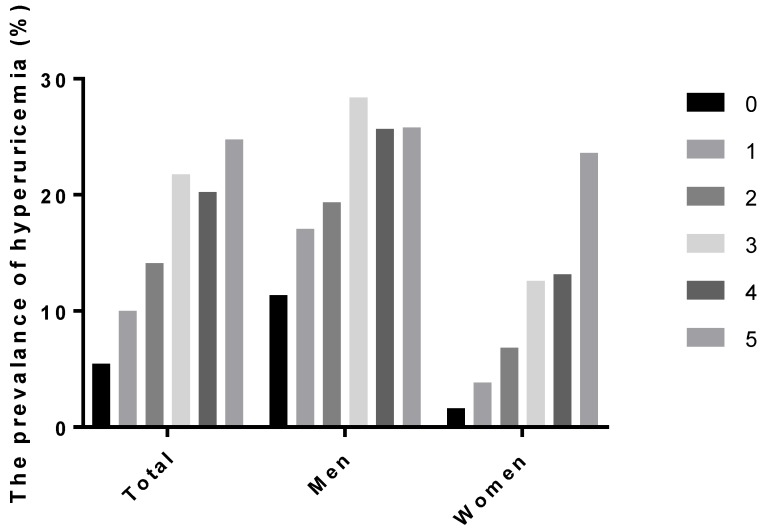
The prevalence of hyperuricemia according to the number of fulfillments of the components of metabolic syndrome.

Figure 1 shows the prevalence of hyperuricemia according to the number that fulfilled five components of MetS. In men, the prevalence of hyperuricemia that met three criteria for MetS was 28.4%, compared to 11.4% of those without any components of MetS. The prevalence of hyperuricemia among women without any MetS components was only 1.6%, but increased to 12.6% when they fulfilled three criteria and further increased up to 23.6% if they met all of the five components. 

### 3.2. Correlation Between sUA Levels with Various Anthropometric and Biochemical Measurements

Correlation between sUA levels with variables according to sex is presented in Table 2. Regardless of sex, sUA levels were positively correlated with BMI, WC, diastolic BP, TG, TC, and LDL-C, and negatively correlated with eGFR and HLD-C. SUA levels showed an inverse correlation with age and fasting plasma glucose (FPG) among men but, in contrast, showed positive correlations in women. 

### 3.3. Risk Factors for Hyperuricemia

Table 3 presents the results of multivariate logistic regression analyses, performed to demonstrate independent risk factors for hyperuricemia. Both MetS and general obesity were significant risk factors for hyperuricemia in the overall population. In the age- and sex-adjusted model (Model 1), the adjusted ORs for having hyperuricemia were 3.06 (95% CI, 2.46–3.80) among subjects with MetS and 2.27 (95% CI, 1.85–2.79) among those with general obesity. These associations remained significant after adjustments for potential confounders, including smoking, alcohol intake, physical activity, and eGFR (Model 2). MetS was significantly associated with an increased risk of hyperuricemia in men (adjusted OR: 2.38, 95% CI: 1.84–3.08) and women (adjusted OR: 4.15, 95% CI: 2.75–6.28). However, we observed sex-based differences regarding the five components of MetS. Raised WC, elevated BP, high TG, and reduced HDL-C were demonstrated as significant risk factors for hyperuricemia in both sexes. Meanwhile, hyperglycemia was associated with hyperuricemia only in women after adjustments. General obesity was also independently associated with hyperuricemia in both sexes with an adjusted odds ratio (OR) of 2.17 (95% CI: 1.76–2.68) in men and 3.80 (95% CI: 2.82–5.12) in women, respectively.

### 3.4. Subgroup Analysis According to the Presence of MetS and General Obesity

Table 4 shows the adjusted ORs (95% CI) for having hyperuricemia, according to the presence of MetS and/or general obesity in subgroups. Subjects without both conditions were designated as a reference group. Age, sex, smoking, alcohol intake, physical activity, and eGFR were adjusted for analyses. Among subjects with a BMI <25 kg/m^2^, MetS was associated with an increased risk of hyperuricemia in both men and women. Among those, males having MetS showed a nearly three times higher risk of hyperuricemia, and females having MetS had a more than four times higher risk of hyperuricemia, compared to those without both conditions. In the overall population, subjects with both general obesity and MetS showed nearly a four-fold increased risk for hyperuricemia. The risk of hyperuricemia in subjects with both conditions has increased up to six-fold among the young age group (aged less than 40 years). Notably, the risk was substantially amplified among women who had both general obesity and MetS, which increased up to a seven-fold risk for hyperuricemia, compared to women without both conditions.

## 4. Discussion

Uric acid is the end product of purine metabolism, and hyperuricemia may lead to complications such as gout, nephropathy, and urinary stones [1,2]. The prevalence of hyperuricemia has increased over the decades worldwide [4,5,6,7] and has become one of the most significant public health issues, given its role as a potential predictor of cardiovascular diseases.

The widely accepted definition of MetS is the clustering of metabolically related cardiovascular risk factors, including hyperglycemia, hypertension, dyslipidemia, and central obesity. Traditionally, insulin resistance has been considered the key pathogenesis of MetS [8,9,10,11,12]. Various studies have shown that hyperuricemia is associated with MetS, insulin resistance, or diabetes, and they have suggested that sUA level is a biomarker for these conditions [19,20,21,22,23,24,25,26,27,28,29,30,31,36,37,38]. According to one prospective study, when compared to subjects with the lowest thirds of sUA levels, those with the upper thirds of sUA levels had a 1.6-fold higher risk for MetS among men, and a more than two-fold higher risk among women [24]. Lv et al. reported a 6% increase of the risk of type 2 DM per 1 mg/dL increment in the sUA level (RR: 1.06, 95% CI: 1.04–1.07) from a meta-analysis of eight prospective cohort studies involving a total of 32,016 individuals [25]. Hyperinsulinemia reduces the renal excretion of uric acid and sodium; thus, hyperuricemia resulting from euglycemic hyperinsulinemia may precede the onset of overt DM [2]. Furthermore, hyperuricemia itself may directly contribute to insulin resistance via inhibition of insulin signaling or as a result of decreased endothelial nitric oxide bioavailability [30,31].

Evidence has revealed that hyperuricemia is associated with adipose tissue and obesity, which is also a well-known risk factor for insulin resistance [43,44,45,46,47]. Tsushima et al. demonstrated from a mouse model that the adipose tissue itself has abundant xanthine oxidoreductase activity that can produce uric acid, which is further augmented in obesity [46]. 

In general, MetS, insulin resistance, and obesity are well-established risk factors for cardiovascular diseases and increased mortality [12,41]. As evidence has accumulated that hyperuricemia is associated with these conditions, it is emerging as one of the potential biomarkers or predicting factors for cardiovascular diseases. 

Results of our study also indicate that the presence of MetS and general obesity is independently associated with hyperuricemia in both men and women. In men, the risk of hyperuricemia has almost doubled with MetS or general obesity after adjustments for age, sex, smoking, alcohol intake, physical activity, and eGFR. Women with MetS or general obesity showed a more prominent increased risk (approximately four-fold after adjustments) of having hyperuricemia. It is plausible that the higher increase in the degree of hyperuricemia accompanied by MetS in women that we observed was related to the higher rates of hyperglycemia and central obesity in hyperuricemic women. Mean serum FPG levels, prevalence of hyperglycemia, and overt DM were higher in women of the hyperuricemic group compared to the normouricemic one. In addition, the difference in proportion of central obesity between the two groups was more prominent in women. 

There is some evidence suggesting sex-based differences in association with sUA levels and MetS, although the mechanisms for this phenomenon still have to be clarified [24,50,51,52,53,54]. According to a recent prospective study, the baseline sUA level predicted new-onset MetS only in women [37]. In addition, several studies have suggested sex-based differences in MetS with other related conditions. Possible mechanisms included a hormonal effect or sex-based differences in insulin sensitivity and body fat composition [55,56,57]. Vigna et al. demonstrated that inflammatory abnormalities were strongly related to sUA and MetS in obese women [50]. Although age and potential confounders were adjusted for the multivariate regression analysis in our study, sex-based differences that we observed might be also driven from unmeasured confounders, given the baseline differences in lifestyle and social factors, including smoking, alcohol, and income levels between men and women. In the meantime, previous studies suggested a more robust association between hyperuricemia and cardiovascular disease among women compared to men [34,35,58]. Taken altogether, it is reasonable to investigate the presence of hyperuricemia in women and men with risk factors. Further research on sex physiology in relation to hyperuricemia and metabolic diseases is required. 

In the subgroup analyses, we estimated the relative risk of having hyperuricemia according to presence of MetS and/or general obesity, compared to the reference group composed of subjects with neither conditions. Among subjects with a normal BMI range (<25 kg/m^2^), the presence of MetS was associated with a three-fold increase of risk for hyperuricemia in men and a four-fold increase in women. Notably, males having MetS despite a normal BMI exhibited an even higher risk for hyperuricemia, compared to an obese male without MetS, often referred to as “metabolically healthy obesity”. Thus, it seems necessary to fully evaluate the presence of hyperuricemia and components of MetS in metabolically deranged men, even with a normal BMI. Our results support the prior suggestions that general obesity defined simply by BMI may not be sufficient to determine true obesity and metabolic risk. For example, the visceral adiposity index (VAI) was recently suggested as a novel sex-specific index for visceral adipose function and a useful predictor for hyperuricemia, irrespective of obesity phenotypes [59].

The presence of concurrent MetS and general obesity was associated with a strikingly increased risk for hyperuricemia in both men and women. Moreover, this increase in risk was nearly six-fold among the young age group and seven-fold among the female subgroup when compared to those without both conditions. It is worthwhile to identify a potential indicator and biomarker to predict metabolic and cardiovascular diseases in the population at risk, and it has been considered that hyperuricemia might be potentially linked to metabolic and cardiovascular diseases. Therefore, these results may suggest that subjects with MetS and general obesity, especially young adults and women, should be screened for the presence of asymptomatic hyperuricemia. 

The limitations of the current study mainly arise from the cross-sectional study design that cannot warrant a causal relationship between hyperuricemia and risk factors. To add, the generalizability might be compromised by selection bias because the KNHANES is a sampling survey. In addition, we were not able to collect more detailed information as this survey did not provide information about the diagnosis of gout and medication usage, including urate-lowering agents and other medications that could affect sUA levels. Despite the limitations, this study result would be useful in respect to public health, because we demonstrated associations between hyperuricemia with both MetS and general obesity according to sex in a large nation-wide representative survey database. 

## 5. Conclusions

In conclusion, both MetS and general obesity were identified as independent risk factors for hyperuricemia in the national representative sample of Korean adults, regardless of sex. MetS conferred a 2.4-fold and 4.2-fold increased risk of hyperuricemia in men and women, respectively. In addition, general obesity posed a 2.2-fold and 3.8-fold higher risk of hyperuricemia in men and women. Hyperuricemia was associated with MetS in spite of a normal BMI range. Young adults and females with both MetS and general obesity carried strikingly higher risks of hyperuricemia compared to those without both conditions. Accordingly, more attention should be paid to the increased risk of hyperuricemia for these populations. 

## Figures and Tables

**Table 1:** Baseline characteristics of study population. A total of 685 individuals were classified as having hyperuricemia among 5591 subjects, based on 2016 Korean National Health and Nutrition Examination Survey (KNHANES).

	Total			Men			Women		
	Hyperuricemia			Hyperuricemia			Hyperuricemia		
	No (*n* = 4906)	Yes (*n* = 685)	*p* Value	No (*n* = 4906)	Yes (*n* = 685)	*p* Value	No (*n* = 4906)	Yes (*n* = 685)	*p* Value
Age (years)	46.7 ± 0.4	45.7 ± 0.8	0.2248	46.4 ± 0.5	42.6 ± 0.8	<0.0001	46.9 ± 0.5	55.2 ± 1.5	<0.0001
Male (%)	46.2(0.7)	75.2(2)	<0.0001						
BMI (kg/m^2^)	23.7 ± 0.1	26 ± 0.2	<0.0001	24.3 ± 0.1	25.9 ± 0.2	<0.0001	23.2 ± 0.1	26.1 ± 0.4	<0.0001
WC (cm)	81.9 ± 0.2	88.8 ± 0.5	<0.0001	85.6 ± 0.3	89.3 ± 0.6	<0.0001	78.7 ± 0.3	87.3 ± 1.0	<0.0001
Systolic BP (mmHg)	117.3 ± 0.3	122.4 ± 0.6	<0.0001	120.2 ± 0.4	122.5 ± 0.7	0.0042	114.8 ± 0.4	122.1 ± 1.4	<0.0001
Diastolic BP (mmHg)	75.6 ± 0.2	79.4 ± 0.5	<0.0001	78.2 ± 0.3	80.8 ± 0.6	<0.0001	73.3 ± 0.3	75.0 ± 0.9	0.0691
FPG (mg/dL)	99.6 ± 0.5	101.2 ± 0.8	0.0805	102.4 ± 0.8	100.2 ± 0.9	0.0661	97.2 ± 0.5	104.2 ± 2.0	0.0008
TG (mg/dL) ^a^	108.92 (106.5–111.39)	157.47 (147.04–168.65)	<0.0001	125.9 (121.8–130.2)	164.3 (151.4–178.3)	<0.0001	96.2 (93.6–98.9)	138.5 (125.2–153.2)	<0.0001
TC (mg/dL)	192.2 ± 0.6	200.2 ± 1.9	<0.0001	190.4 ± 0.9	199.4 ± 2.4	0.0006	193.7 ± 0.8	202.7 ± 3.0	0.0039
HDL-C (mg/dL)	52 ± 0.2	46 ± 0.5	<0.0001	48.3 ± 0.3	44.6 ± 0.6	<0.0001	55.2 ± 0.3	50.4 ± 1.0	<0.0001
LDL-C (mg/dL)	114.9 ± 0.6	118.6 ± 1.5	0.0133	113.2 ± 0.8	118.0 ± 1.8	0.014	116.2 ± 0.7	120.6 ± 2.6	0.1066
eGFR (ml/min/1.73 m^2^)	97.8 ± 0.5	87.4 ± 0.9	<0.0001	95.2 ± 0.7	89.6 ± 1.0	<0.0001	99.9 ± 0.6	80.7 ± 1.8	<0.0001
Cigarette smoking (%)			<0.0001			0.548			0.0053
Never-smoker	61.2(0.9)	43.7(2.3)		27.6(1.2)	30.0(2.7)		90(0.8)	85.3(2.6)	
Ex-smoker	17.9(0.6)	23.8(2)		33.4(1.2)	30.5(2.5)		4.6(0.5)	3.4(1.3)	
Current-smoker	20.9(0.9)	32.5(2.1)		38.9(1.5)	39.5(2.7)		5.5(0.6)	11.3(2.4)	
Alcohol intake (%)			<0.0001			0.2756			0.0291
Non drinker	23.4(0.8)	19.2(1.7)		13.7(0.9)	13.2(1.7)		31.7(1.1)	37.2(3.6)	
Light drinker	68.2(0.9)	65.2(2.1)		70.6(1.3)	67.5(2.5)		66.3(1.1)	58.4(3.7)	
Moderate-to-heavy drinker	8.4(0.5)	15.6(1.6)		15.8(1.0)	19.3(2.1)		2.1(0.3)	4.4(1.6)	
Physically active (%)	47.8(1.1)	50.5(2.5)	0.2831	51.4(1.5)	53.1(3)	0.6036	44.7(1.2)	43.0(3.6)	0.6428
Employed (%)	63.5(1)	63.1(2.3)	0.8488	75.6(1.3)	72.8(2.7)	0.3076	53.3(1.2)	34.6(3.7)	0.0671
Low-income (%)	15.4(1)	18.1(2.1)	0.1218	13.9(1.2)	14.2(2.2)	0.8755	16.7(1.1)	30.1(3.8)	<0.0001
Diabetes (%)	10.6(0.5)	11(1.4)	0.8044	12.5(0.8)	7(1.2)	0.0017	9.0(0.7)	22.9(3.5)	<0.0001
Hypertension (%)	26.2(0.8)	39.6(2.2)	<0.0001	30.6(1.3)	36.3(2.3)	0.0266	22.4(1.0)	49.6(4.3)	<0.0001
**MetS, yes (%)**	28.5(0.9)	51.8(2.3)	<0.0001	32.5(1.3)	52.1(1.1)	<0.0001	48.5(2.5)	61.6(4.0)	0.0028
Raised WC ^b^	26.8(0.9)	48.2(2.3)	<0.0001	28.9(1.3)	43.1(2.7)	<0.0001	25(1.1)	63.5(3.8)	<0.0001
Elevated BP	36.1(0.9)	54.1(2.4)	<0.0001	44.1(1.3)	53.2(2.5)	0.0015	29.2(1.2)	57(4.4)	<0.0001
Hyperglycemia	32.2(0.9)	44(2.2)	<0.0001	39.2(1.4)	40.3(2.6)	0.7118	26.2(1.0)	55.3(3.9)	<0.0001
Elevated TG	34.1(0.8)	57.3(2.4)	<0.0001	42.1(1.3)	59.2(2.7)	<0.0001	27.2(1.1)	51.5(4.2)	<0.0001
Reduced HDL-C	36(0.9)	44.8(2.1)	<0.0001	30.5(1.2)	40.3(2.3)	<0.0001	40.7(1.1)	58.7(4.1)	<0.0001
**General obesity ^c^, yes (%)**	32.6(1)	55.1(2.4)	<0.0001	39.2(1.4)	53.4(2.8)	<0.0001	26.9(1.1)	60.4(4.1)	<0.0001

^a^ Geometric means. Log transformation of variables was performed to calculate *p* values. ^b^ Defined by the ethnic-specific values for waist circumference (WC), ≥90 cm in men or ≥85 cm in women. ^c^ General obesity was defined by a body mass index (BMI) ≥25 kg/m^2^. Values are presented as means with standard deviation (SD) or %. *P*-values calculated by student’s t-test for continuous data or X2 test for categorical data. Abbreviations: BMI, body mass index; WC, waist circumference; BP, blood pressure; FPG, fasting plasma glucose; TG, triglycerides; TC, total cholesterol; HDL-C, HDL-cholesterol; LDL-C, LDL-cholesterol; eGFR, estimated glomerular filtration rate; MetS, metabolic syndrome.

**Table 2:** Correlation coefficients of serum uric acid levels with various anthropometric and biochemical parameters.

	Total		Men		Women	
	*r*	*p* Value	*r*	*p* Value	*r*	*p* Value
Age	−0.06793	<0.0001	−0.15000	<0.0001	0.08851	0.0001
eGFR	−0.22997	<0.0001	−0.13437	<0.0001	−0.30527	<0.0001
BMI	0.27971	<0.0001	0.21723	<0.0001	0.24475	<0.0001
WC	0.35672	<0.0001	0.19747	<0.0001	0.24003	<0.0001
Systolic BP	0.14621	<0.0001	0.02752	0.1839	0.10715	<0.0001
Diastolic BP	0.21096	<0.0001	0.09230	0.0006	0.07835	0.0005
FPG	0.01891	0.1924	−0.10723	0.0006	0.0587	0.0084
TG ^a^	0.29459	<0.0001	0.19604	<0.0001	0.20503	<0.0001
TC	0.07116	<0.0001	0.11839	<0.0001	0.08494	<0.0001
HDL-C	−0.26125	<0.0001	−0.15671	<0.0001	−0.11712	<0.0001
LDL-C	0.03867	0.0211	0.08497	0.0003	0.05192	0.01

^a^ Log transformation of variables was performed to calculate *p* values. BMI: Body mass index; WC: Waist circumference; BP: Blood pressure; FPG: Fasting plasma glucose; TG: Triglycerides; TC: Total cholesterol; HLD-C: HLD-cholesterol; LDL-C: LDL-cholesterol; eGFR: Estimated glomerular filtration rate.

**Table 3:** Multivariate-adjusted odds ratios (OR) of the association of hyperuricemia with metabolic syndrome (MetS), various components of the metabolic syndrome, and general obesity (defined by a BMI ≥25 kg/m^2^).

	Total (*n* = 5591)		Men (*n* = 2429)		Women (*n* = 3162)	
	Model 1 OR (95% CI)	Model 2 OR (95% CI)	Model 1 OR (95% CI)	Model 2 OR (95% CI)	Model 1 OR (95% CI)	Model 2 OR (95% CI)
**Raised WC**	2.577 (2.116, 3.139)	2.521 (2.067,3.076)	1.935 (1.525, 2.457)	1.82 (1.436, 2.308)	4.429 (3.155, 6.218)	4.895 (3.381, 7.086)
**Elevated BP**	2.177 (1.749, 2.709)	2.067 (1.648,2.592)	1.898 (1.495, 2.411)	1.807 (1.404, 2.326)	2.373 (1.403, 4.012)	2.290 (1.373, 3.820)
**Hyperglycemia**	1.576 (1.290, 1.926)	1.584 (1.269,1.979)	1.290 (0.997, 1.670)	1.300 (0.983, 1.720)	2.733 (1.930, 3.870)	2.729 (1.881, 3.961)
**Elevated TG**	2.324 (1.885, 2.865)	2.364 (1.901,2.939)	2.160 (1.697, 2.749)	2.190 (1.699, 2.824)	2.190 (1.472, 3.260)	2.350(1.610, 3.430)
**Reduced HDL-C**	1.747 (1.441, 2.118)	1.758 (1.445,2.139)	1.737 (1.392, 2.168)	1.789 (1.414, 2.264)	1.612 (1.126, 2.308)	1.594 (1.102, 2.306)
**Metabolic syndrome**	3.061 (2.462, 3.804)	2.917 (2.309,3.685)	2.502 (1.982, 3.158)	2.380 (1.842, 3.075)	4.135 (2.751, 6.215)	4.153 (2.747, 6.278)
**General obesity**	2.268 (1.846, 2.786)	2.626 (2.213,3.116)	1.746 (1.360, 2.242)	2.170 (1.756, 2.681)	3.580 (2.510, 5.106)	3.801 (2.823, 5.117)

Model1: Adjusted for age and sex. Mode2: Adjusted for age, sex, smoking, alcohol intake, physical activity, eGFR. BMI: Body mass index; WC: Waist circumference; BP: Blood pressure; TG: Triglycerides; HDL-C: HDL-cholesterol.

**Table 4:** The odds ratios for the presence of hyperuricemia according to presence of metabolic syndrome and general obesity.

	Total (*n* = 5591)	Age			Sex	
		<40 (*n* = 1663)	40–64 (*n* = 2555)	65- (*n* = 1373)	Men (*n* = 2429)	Women (*n* = 3162)
	OR (95% CI)	OR (95% CI)	OR (95% CI)	OR (95% CI)	OR (95% CI)	OR (95% CI)
GO (–) MetS (–)	1 (reference)	1 (reference)	1 (reference)	1 (reference)	1 (reference)	1 (reference)
GO (–) MetS (+)	3.207 (2.278, 4.516)	2.509 (0.944, 6.666)	3.165 (1.983, 5.051)	2.958 (1.787, 4.898)	2.922 (1.927, 4.431)	4.531 (2.758, 7.444)
GO (+) MetS (–)	2.203 (1.586, 3.059)	2.424 (1.525, 3.853)	1.666 (0.993, 2.794)	2.671 (1.390, 5.132)	1.601 (1.094, 2.341)	4.595 (2.714, 7.781)
GO (+) MetS (+)	3.906 (2.996, 5.093)	5.971 (3.662, 9.736)	3.015 (2.015, 4.511)	3.929 (2.483, 6.218)	2.895 (2.116, 3.960)	7.238 (4.555, 11.501)

Age, sex, smoking, alcohol intake, physical activity, and eGFR were adjusted. GO: General obesity (defined by BMI ≥25 kg/m^2^); MetS: Metabolic syndrome.
